# One World, One Health: Zoonotic Diseases, Parasitic Diseases, and Infectious Diseases

**DOI:** 10.3390/healthcare12090922

**Published:** 2024-04-29

**Authors:** Giovanna Deiana, Antonella Arghittu, Marco Dettori, Paolo Castiglia

**Affiliations:** 1 Department of Biomedical Sciences, University of Sassari, 07100 Sassari, Italy; 2 Medical Management, Hygiene, Epidemiology and Hospital Infection, University Hospital of Sassari, 07100 Sassari, Italy; madettori@uniss.it (M.D.); paolo.castiglia@uniss.it (P.C.); 3 Department of Medicine, Surgery and Pharmacy, University of Sassari, 07100 Sassari, Italy; aarghittu@uniss.it

When we take into account how the boundaries between human, animal, and environmental health are inextricably linked and increasingly intertwined, it comes as no surprise that the One Health approach has assumed an unprecedented level of importance over the past decade [1]. This holistic, integrated systems-based approach strongly advocates for the interconnectedness of human, animal, and environmental health, putting the emphasis on their mutually dependent well-being [2,3,4,5].

At the heart of the One Health approach, the environment plays a pivotal role, acting as a bridge between human and animal health and influencing the occurrence and spread of diseases [6,7]. The World Health Organization (WHO) estimates that one in every four deaths globally can be attributed to environmental factors. These factors can contribute to a wide range of diseases and conditions, disproportionately affecting vulnerable groups such as children and the elderly [8,9]. Thus, it is essential to implement a unified and cross-sectoral global strategy to improve the quality of water, soil, air, and indoor environments, thereby addressing the impacts on ecosystems and mitigating the health effects attributable to environmental risk factors [10,11,12,13,14].

Human activities have an enormous impact on natural environments, i.e., overuse and/or contamination of natural resources threatens ecosystems and affects both human and animal health. For example, contamination of water bodies with harmful chemicals is closely associated with the spread of waterborne diseases [15,16,17,18,19]. Adverse events, such as habitat destruction, climate change, and biodiversity loss, disrupt ecological balances, facilitating the transmission of zoonotic and parasitic diseases, even from animals to humans. As such, deforestation and the encroachment of human activities into wildlife habitats greatly increase the likelihood of disease spillover events. Similarly, climate change alters the geographic range of disease vectors (e.g., mosquitoes), thereby expanding the reach of diseases like malaria and dengue fever [20,21,22].

The One Health approach recognizes that maintaining environmental integrity is crucial for controlling and preventing zoonotic and parasitic diseases [23,24,25,26]. The recent COVID-19 pandemic, as well as avian influenza and Ebola, is a stark reminder of how a disease originating in animals can have a global impact on human health and societies as a whole [27,28]. In fact, one of the most recent Ebola virus disease outbreaks affected over 28,600 cases from 2014 to 2016 in West Africa, with additional cases reported in other countries (Italy, Spain, the United Kingdom, and the United States). Meanwhile, the H5N1 bird flu has been extensively detected in the U.S., with 9253 wild birds and 90,604,876 poultry testing positive in the latest annual survey, posing a serious risk to human health [29,30]. The transmission dynamics of such diseases are often multifaceted: increased urbanization pushes humans into previously unpopulated areas, climate change alters the habitats of disease-carrying species, and global travel and trade facilitate the rapid spread of pathogens [31,32,33]. In particular, 75% of emerging infectious human diseases have an animal origin, and 60% of pathogens that cause human diseases originate from domestic animals or wildlife. In 2022, the most frequently reported zoonotic diseases in humans within the EU were campylobacteriosis, with 137,000 cases, and salmonellosis, with 65,000 cases. Yersiniosis was the third most reported zoonosis, followed by infections caused by Shiga toxin-producing *Escherichia coli* (STEC) and *Listeria monocytogenes* [34,35].

Parasitic diseases, while often less sensationalized, pose a significant and persistent threat to global health [36,37]. Malaria, schistosomiasis, and leishmaniasis affect millions worldwide, particularly in tropical and subtropical regions [38,39]. For instance, in 2020, there were an estimated 241 million cases of malaria globally, leading to approximately 627,000 deaths. Most of these deaths occurred among children in sub-Saharan Africa. Similarly, the WHO Foodborne Disease Burden Epidemiology Reference Group estimated that echinococcosis, whose prevalence among livestock varies from 20–95% in hyperendemic areas, causes up to 19,300 deaths in humans and about 871,000 disability-adjusted life years (DALYs) globally each year. Additionally, prevalence surveys indicate that about 10–30% of dogs and slightly fewer cats are infected with *Giardia*, with younger animals showing higher rates. Whereas, in small ruminants like sheep and goats, the infection rates generally range from 20% to 25%, although they can vary from less than 10% to over 40%, with cattle showing similar patterns [40,41,42]. Tackling these diseases requires not only medical intervention but also improvements in living conditions and education regarding prevention methods [43,44]. Infectious diseases, whether bacterial, viral, or parasitic, continue to evolve and adapt, presenting ongoing challenges to public health [45,46]. The clinical relevance of these diseases pertains to their impact on individual patients and on public health systems globally. As for individuals, infectious diseases can cause a wide range of symptoms and outcomes, from mild, self-limiting illnesses to severe, life-threatening conditions, and can lead to complications, including organ damage, secondary infections, and long-term disability. Diseases like HIV/AIDS, tuberculosis, and malaria have high mortality rates and are major causes of morbidity worldwide. Moreover, infectious diseases contribute significantly to healthcare costs due to hospitalizations, treatments, lost productivity, and premature death. Concurrently, the overuse of antibiotics has led to the rise of drug-resistant strains of bacteria, while changes in human behavior and the environment may lead to the emergence of new viruses [47,48,49,50].

According to the WHO, a collaborative, cross-disciplinary approach is essential in the fight against these health threats. Such an approach involves, among other things, strengthening health systems, improving surveillance and response capabilities, and investing in research and development for new treatments and vaccines. It is important to note that efforts must also focus on preventing outbreaks at their source, which means protecting natural habitats, regulating wildlife trade, and improving animal health [51,52]. Consequently, the WHO, in collaboration with the Food and Agriculture Organization of the United Nations (FAO), the United Nations Environment Program (UNEP), and the World Organization for Animal Health (WOAH), has implemented a joint action plan through an integrated approach in which knowledge and skills are oriented towards a culture of transversality, which translates into the One Health Action Plan 2022–2026 [53].

In this context, food traceability plays a pivotal role in the control and prevention of infectious diseases transmitted through the food supply. Effective traceability systems help in identifying the origin of contamination more rapidly and accurately, facilitating targeted recalls, preventing further consumption of tainted products, and effectively limiting the spread of foodborne illnesses. In the broader context of public health, food traceability is not just a tool for crisis management but a foundational aspect of preventive health policies, helping to mitigate the risk of large-scale foodborne outbreaks and enhancing the overall safety of the food supply [54,55]. The critical importance of these systems was starkly highlighted by the mad cow disease outbreak (Bovine Spongiform Encephalopathy, BSE). This crisis revealed that prions could be transmitted to herbivores through non-cannibalistic routes, specifically via meat-based feeds. The incident accelerated the rapid development of stringent traceability and tracking systems aimed at preventing such mistakes from recurring [56,57].

The One Health concept becomes even more pertinent when considering the complex challenges of the 21st century. Globalization and the rapid pace of urbanization, significantly increasing the interconnection between people, animals, and goods across borders, have exacerbated the spread of diseases. In response, the development of technologies like genomic sequencing, AI, and remote sensing can improve disease surveillance, track environmental changes, and further our understanding of the complex interactions between human, animal, and environmental health [58,59,60,61,62,63].

Fortunately, climate-related concerns have now moved to the forefront of global public consciousness, profoundly shaping political, social, and economic narratives. The escalating awareness of climate change, underscored by alarming scientific findings and the direct consequences of extreme weather phenomena, a rise in sea level, and biodiversity loss, has sparked an unparalleled sense of urgency worldwide. This heightened public concern has not only fueled innovation in the renewable energy sector but has also played a crucial role in driving the implementation of more rigorous environmental policies across many nations. In essence, the influence of climate issues on public opinion is forging a new paradigm, one that emphasizes sustainability and a shared commitment to our planet’s well-being [64,65,66].

In summary, the One Health concept is not merely a theoretical model but also a practical necessity in our interconnected world. It calls for a collaborative, cross-sectoral, and transdisciplinary approach, integrating human, animal, and environmental health. As we face an increasing number of zoonotic, parasitic, and infectious diseases, governments, international organizations, health professionals, and communities worldwide must embrace and integrate the One Health approach to safeguard the health of our planet and its inhabitants [67,68,69].
